# Improving the Quantification of the Lateral Geniculate Nucleus in Magnetic Resonance Imaging Using a Novel 3D-Edge Enhancement Technique

**DOI:** 10.3389/fncom.2021.708866

**Published:** 2021-12-03

**Authors:** Mikhail Lipin, Jean Bennett, Gui-Shuang Ying, Yinxi Yu, Manzar Ashtari

**Affiliations:** ^1^Department of Ophthalmology, Perelman School of Medicine, University of Pennsylvania, Philadelphia, PA, United States; ^2^Center for Preventative Ophthalmology and Biostatistics, Department of Ophthalmology, Perelman School of Medicine, University of Pennsylvania, Philadelphia, PA, United States

**Keywords:** segmentation, brain morphometry, MRI, noise immunity, partial volume effect, LGN

## Abstract

The lateral geniculate nucleus (LGN) is a small, inhomogeneous structure that relays major sensory inputs from the retina to the visual cortex. LGN morphology has been intensively studied due to various retinal diseases, as well as in the context of normal brain development. However, many of the methods used for LGN structural evaluations have not adequately addressed the challenges presented by the suboptimal routine MRI imaging of this structure. Here, we propose a novel method of edge enhancement that allows for high reliability and accuracy with regard to LGN morphometry, using routine 3D-MRI imaging protocols. This new algorithm is based on modeling a small brain structure as a polyhedron with its faces, edges, and vertices fitted with one plane, the intersection of two planes, and the intersection of three planes, respectively. This algorithm dramatically increases the contrast-to-noise ratio between the LGN and its surrounding structures as well as doubling the original spatial resolution. To show the algorithm efficacy, two raters (MA and ML) measured LGN volumes bilaterally in 19 subjects using the edge-enhanced LGN extracted areas from the 3D-T1 weighted images. The averages of the left and right LGN volumes from the two raters were 175 ± 8 and 174 ± 9 mm^3^, respectively. The intra-class correlations between raters were 0.74 for the left and 0.81 for the right LGN volumes. The high contrast edge-enhanced LGN images presented here, from a 7-min routine 3T-MRI acquisition, is qualitatively comparable to previously reported LGN images that were acquired using a proton density sequence with 30–40 averages and 1.5-h of acquisition time. The proposed edge-enhancement algorithm is not limited only to the LGN, but can significantly improve the contrast-to-noise ratio of any small deep-seated gray matter brain structure that is prone to high-levels of noise and partial volume effects, and can also increase their morphometric accuracy and reliability. An immensely useful feature of the proposed algorithm is that it can be used retrospectively on noisy and low contrast 3D brain images previously acquired as part of any routine clinical MRI visit.

## Introduction

The lateral geniculate nucleus (LGN) is a small wedge-shaped ventral area at the termination of the optic tract on each side of the brain. The LGN is the relay center and main hub for visual processing, connecting the output of the retina to the primary visual cortex, and playing an early gatekeeper role in the control of visual attention and awareness (Kastner et al., 2006). In addition to the afferent connections, the LGN receives strong efferent connections from the primary visual cortex that modulate attentional function and the coordination of cortical regions (Halassa and Kastner, 2017).

The crucial role of LGN in visual processing has been the focus of investigations into how various visual and brain disorders may affect the LGN’s morphology and function (Garey and de Courten, 1983; Bush and Allman, 2004). For example, the LGN was found to be enlarged in patients with mood disorders, but not in patients with schizophrenia (Selemon and Begovic, 2007; Dorph-Petersen et al., 2009). The LGN has been reported to be significantly smaller in volume and differed in shape in patients with dyslexia (Giraldo-Chica et al., 2015; Müller-Axt et al., 2017; Giraldo-Chica and Schneider, 2018). The LGN volume has also been reported to be reduced in patients with one eye (Moro et al., 2015; Wong et al., 2018), in patients with neuromyelitis optica spectrum disorders (Papadopoulou et al., 2019a,b), glaucoma (Dai et al., 2011; Hernowo et al., 2011; Zhang Y.Q. et al., 2012; Chen et al., 2013; Lee et al., 2014; Wang et al., 2015, 2016; Schmidt et al., 2018), albinism (McKetton et al., 2014; Grigorian et al., 2016), patients with hemianopia (Bridge et al., 2011), and patients with Parkinson’s disease (Bohnen et al., 2019). The volume of the LGN has also been shown to be significantly affected by the normal aging process (Li et al., 2012).

To date, Magnetic Resonance Imaging (MRI) is an indispensable method for visualizing not only gross neuroanatomy but also deep-seated gray matter nuclei such as the LGN, *in vivo* (Fujita et al., 2001; Li et al., 2012; Aldusary et al., 2019). However, as with the imaging of other structures, there are two main issues that severely compromise the visualization, delineation and accuracy of LGN volume measurements: first is the area’s low contrast-to-noise ratio (CNR), and second is the problem of partial volume uncertainty. At times, the low CNR and/or high partial volume effect/artifact is so severe that the LGN is only qualitatively assessed (Bridge et al., 2011) with no quantification. The low CNR in LGN imaging results from the low contrast of the LGN with respect to its surrounding tissue composition (e.g., white matter, gray matter, CSF) (Horton et al., 1990). The LGN image contrast can be enhanced by optimizing the MRI sequences, for example, using white matter nulled sequences (Aldusary et al., 2019) to enhance gray matter structures or to increase CNR by averaging over repetition time (TR) (McKetton et al., 2015). However, increasing LGN visibility by optimizing MR sequence parameters often occurs at the higher cost of increased acquisition time and possibility of greater subject motion.

The CNR of the LGN images can also be increased by using a high magnetic field, such as 7T MRI systems (Lee et al., 2014; Schmidt et al., 2018; Aldusary et al., 2019). Although high magnetic field systems (e.g., 7 Tesla) improve CNR and offer better LGN visibility, they are rarely used in routine clinical imaging for patient diagnostic evaluations. Therefore, there still remains a great need for the improvement of LGN visibility on the 3D T1 weighted images that are a major component of the routine clinical protocols of radiological evaluations.

The image noise can be further reduced by post-processing. The most efficient state-of-the-art methods of denoising MR images are based on the non-local means (NLM) algorithm which counteracts noise using redundancy found in natural images (Coupé et al., 2008; Buades et al., 2010; Sutour et al., 2014). The NLM algorithm finds and averages similar patches extracted around each pixel rather than averaging nearby pixels. This method effectively removes the noise from smooth areas and repetitive textures with a size of 7 × 7 × 7 mm^3^ or larger (Manjón et al., 2010a; Coupé et al., 2013), but might not work for denoising small, irregular, or inhomogeneous structures such as the LGN, and is prone to false detections of similar patches at high image noise (Sutour et al., 2014).

**FIGURE 1:**
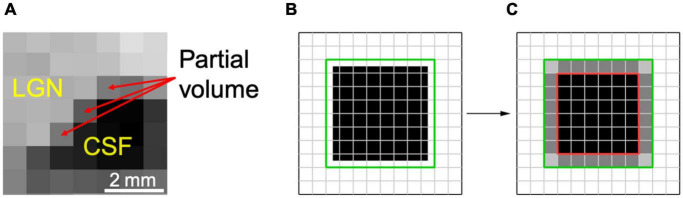
Partial volume uncertainty. **(A)** A segment of the right LGN in coronal orientation depicting partial volume pixels by red arrows. **(B)** The LGN is estimated as a cubical object in a 7 × 7 × 7 voxel box (black). The green line demarcates a co-centered box of 8 × 8 × 8 voxels or total of 512 cube voxels. **(C)** Full volume is shown in black within a cube of 6 × 6 × 6 voxels, encompassing 216 voxels (inside a red box). The partial volume is represented as gray voxels between the red and green boxes, amounted to 296 voxels. Depending on which partial volume voxels are counted, the measured volume of the object would be between 216 and 512 voxels.

Partial volume artifact occurs when a structure is not within the imaging building block of image resolution (pixel, voxel) in its entirety, because of the limited resolution of the imaging system. This will result in signal averaging of the structure of interest with other adjacent or surrounding structures. These issues surrounding the uncertainty of a pixel, to be accounted or not accounted for as part of LGN volume, primarily occurs at the border (edge) of the LGN and its surrounding tissue. Such partial volume induced edge artifact is easily shown in the example below where the LGN borders cerebral spinal fluid (CSF). As shown in Figure 1A, the pixels with light gray intensity primarily belong to the LGN, whereas the pixels with dark black intensity are occupied by CSF. The border pixels between the LGN and CSF, denoted by the red arrows, point to voxels that belong neither entirely to LGN nor CSF, causing the well-known phenomenon of partial volume. Thus, the signal intensity of the LGN border voxels are varied from the voxels that are encompassing the LGN entirely, making accurate assignment of these voxels to the LGN difficult. This ambiguity may be resolved by setting a threshold exactly to the mean value of the voxel intensities of LGN and non-LGN tissue. However, due to the intrinsic inhomogeneity within the LGN structure (McKetton et al., 2015; Müller-Axt et al., 2020), variability in the type of the neighboring tissue (gray matter, white matter, CSF), and elevated noise (Horton et al., 1990), the best value of the threshold is difficult to calculate. Setting the threshold for a voxel intensity to match the measured LGN volume to some previously obtained values such as LGN signals from the imaging of the fixed brain tissue (Renauld et al., 2016) is prone to underestimation. The LGN volume in post-mortem fixed tissue could be half of *in vivo* LGN volume measurements (McKetton et al., 2015).

As shown in Figures 1B,C, the magnitude of partial volume artifact and its effect on errors associated with LGN volume assessment can be demonstrated by estimating the LGN as a cube-shaped object (Castaldi et al., 2016). Figure 1B shows LGN as a cubical object of a size of 7 × 7 × 7 voxels (in black) with an isotropic resolution of 0.8 mm and a true volume of 175.6 mm^3^. Figure 1C demarcates the LGN surrounding environment as a green box, estimated with a co-centered box of 8 × 8 × 8 voxels and a total of 512 voxels. The partial volume is shown in Figure 1C as gray voxels surrounding a 6 × 6 × 6 cube (red box) with a volume of 216 voxels, or 110.6 mm^3^. Assuming all the edge voxels contribute to the partial volume, it would result to 296 voxels (gray voxels between the red and green boxes), or 151.6 mm^3^. Depending on whether no or all partial volume voxels are accounted for as part of LGN volume, the measured volume of the LGN would be between 110.6 and 262.1 mm^3^ (or ∼2.4-fold), thus making the partial volume uncertainty the major source of the variability in the LGN volume measurements. For example, the range of human LGN volume measured in proton density MR images of 100–240 mm^3^ (Bürgel et al., 1999) or 112–276 mm^3^ (Grigorian et al., 2016) is in good agreement with our estimate of the range of LGN volume of 110.6–262.1 mm^3^. The partial volume uncertainty can generally be decreased by increasing image resolution (McKetton et al., 2015; Aldusary et al., 2019), but that often results in lower CNR and, therefore, a much noisier image. In addition, brain motion due to physiological noise, such as pulsation and respiration as well as head motion (McKetton et al., 2015) would be more noticeable when using a voxel size smaller than the magnitude of the brain motion.

The only way to reduce the partial volume uncertainty is dividing the MRI image voxel into smaller subvoxels, each of which either belong or not belong to the LGN, is through image upsampling (increasing resolution). To date, there are two methods of the image upsampling that have been shown to be useful in MRI—non-local means (NLM) and recently developed machine learning. The advent of machine learning techniques has made possible super-resolution reconstruction of the low-resolution images, guided by previously scanned high-resolution datasets (Chun et al., 2019). The method requires training of the neural network with reference high-resolution images. Unfortunately, the reference high-resolution images are not always available. In any case, high-resolution reconstruction of disease-affected brain structures might be biased toward the shape and size of healthy brain structures utilized in the machine learning process. There are also numerous sophisticated modifications of NLM based on the image self-similarity that have proven to be extremely effective for high-resolution image reconstruction (Protter et al., 2009; Manjon et al., 2010b; Manjón et al., 2010a; Zhang K. et al., 2012; Coupé et al., 2013; Jafari-Khouzani, 2014; Mahmoudzadeh and Kashou, 2014). However, these NLM methods are mainly designed for large-scale images with high redundancy and are highly unlikely to be effective for small brain structures, such as the LGN, with poor self-similarity and low CNR. While the upsampling is a necessary step in reducing the partial volume uncertainty, the methods that are effective for large scale images should also be effective in improving the image quality of small heterogeneous brain structures with high image noise levels. In addition, for comparison purposes, the method should be applicable to previously obtained images of patients using a routine clinical protocol to assess the effect of any disease progression or effectiveness of any intervention on the morphological changes of the LGN over time.

Here, we propose a novel method of MR image processing that greatly improves the visibility of the border between LGN and non-LGN tissue by fitting a local edge to each voxel and its immediate neighboring voxels, creating an improved upsampled LGN image with enhanced contrast. The upsampling would counteract the partial volume uncertainty and the edge fitting process results in a higher CNR and images that are more reliable for LGN volume measurements. However, the proposed method is not designed for recovering the LGN’s fine internal details that may be low resolution, and can be used only to trace the outline of the LGN.

## Materials and Equipment

Magnetic Resonance Imaging scans were conducted at the Children’s Hospital of Philadelphia (CHOP) on a research-dedicated 3T Siemens Verio system equipped with a 32-channel head coil.

## Methods

### Study Participants

The demographic characteristics of the subjects are presented in Table 1. All subjects were healthy volunteers, recruited through advertisements and fliers distributed in libraries, doctors’ offices, and community centers by a research coordinator. Exclusion criteria for all subjects included a documented history of developmental delay, a history of substance abuse/dependence, and any history of neurological disorder. The Institutional Review Board at the Children’s Hospital of Philadelphia and the University of Pennsylvania granted approval for this study. After the study was explained to subjects and their parents, written assent and informed consent were obtained.

**TABLE 1:** Demographic characteristics of the study participants.

The number of subjects	19
Male/Female	10/9
Age, median (years)	11
Age, range (years)	8–32
Age, mean ± SD (years)	14.3 ± 7.8
Caucasian/Non-Caucasian	17/2
Dextral/Non-Dextral	16/3

In total, 19 subjects took part in the study, of which ten were males and nine females. The mean age of the participants was 14.3 ± 7.8 years (mean ± SD), the median age was 11 years, and the age range was between 8 and 32 years. Two participants were non-Caucasian, and 17 were Caucasian. Sixteen of the participants were dextral and 3 were non-dextral.

### Magnetic Resonance Imaging

All MRI scans were carried out by a single operator and monitored to be free of artifacts at the time of acquisition. Each subject’s head was secured in the head coil using foam padding to reduce motion. All subjects underwent MRI and a T1 weighted 3D magnetization prepared rapid acquisition gradient echo sequence was obtained with inversion preparation pulse, repetition time of 2080 ms, echo time of 2.54 ms, matrix size of 320 × 320, field of view of 256 × 256 mm^2^, number of slices of 192, slice thickness of 0.8 mm, inversion time of 1200 ms with flip angle of 8°, number of excitations of 1, integrated parallel acquisition techniques factor of 2, and acquisition time of 7:04 min.

### Image Processing

The LGN was visually identified on T1-weighted 3D- MPRAGE images in the sagittal and coronal projections using anatomical landmarks (Korsholm et al., 2007; Li et al., 2012; Aldusary et al., 2019), and its approximate central coordinates were determined using the ITK-SNAP software.^1^ Then, a region of interest (ROI) of a size of 22 × 22 × 22 voxels (with a spatial resolution of 0.8 mm), containing the LGN in its entirety, was extracted with the help of the built-in c3d software. The extracted ROI was transferred to the IgorPro software (WaveMetrics, Oregon^2^) in order to improve the visibility of the LGN using the custom made 3D-edge enhancement algorithm that is the subject of this report. The shape of the LGN in the edge-enhanced image was approximated as a polyhedron. Local features of the polyhedron, such as the face, edge, and vertex, were represented by one plane, the intersection of two planes, and the intersection of three planes, respectively. The edge enhancement was based on finding the local feature out of a set of predefined units of a size of 3 × 3 × 3 voxels that fits an image voxel and its immediate neighbors. To represent the edge-enhanced image with double of the native spatial resolution (0.4 mm), a set of 6 × 6 × 6 voxel units was used.

**FIGURE 2:**
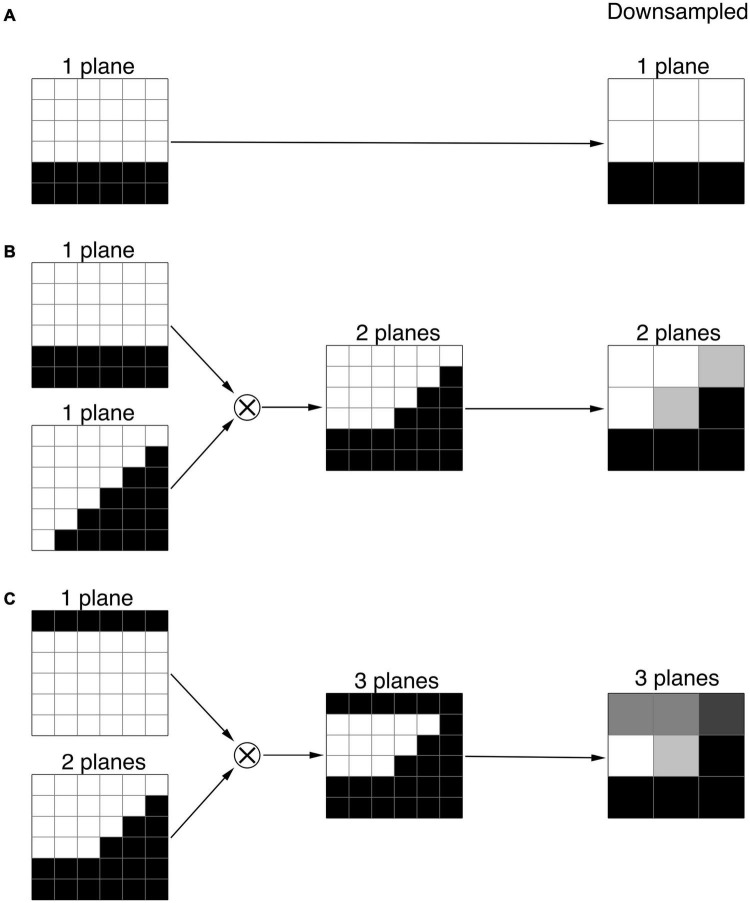
Examples of the one, two, and three plane units. **(A)** 1-plane units made of 6 × 6 × 6 voxel cube crossed by a horizontal plane. **(B)** 2-plane units constructed by the products of two 1-plane units. **(C)** 3-plane units made of the products of 1 and 2-plane units. The far column demonstrates the corresponding downsampled 1, 2, and 3 plane units.

The set of 6 × 6 × 6 voxel units was composed of one, two, and three-plane edges. A series of one plane edges were constructed with 6 × 6 × 6 voxel cubes crossed by a single plane, which formed a continuous edge with values of 0 or 1, at all possible angles and locations. The voxel intensity was assigned a value of 0 when the mean value of the edge within the voxel was less than 0.5, and a value of 1 otherwise. An example of such single plane unit is shown in the top panel of Figure 2. Though the number of all possible angles and locations of a plane is infinite, the number of unique single plane edges turned out to be limited to 109 due to the limited number of voxels in the 6 × 6 × 6 voxel cube (216 voxels). Two-plane units were constructed with all possible products of one-plane units. An example of a two-plane unit is presented in the middle panel of Figure 2. In practice, the number of all possible two-plane units is extremely large, more than ten thousand. To reduce the number of two-plane units, the units with a variance less than 0.19, which represent the edges at the very periphery, were excluded, arriving at 768 two-plane units. The number of two-plane units was reduced to decrease the processing time. The three-plane units were made of all possible combinations obtained from products of one and two-plane units. An example of a three-plane unit is presented in the bottom panel of Figure 2. Using the above-mentioned elimination process we arrived at 34048 number for three-plane units.

As mentioned earlier, the voxel resolution of the 6 × 6 × 6 cube (0.4 mm isotropic) was twice that of the original LGN containing ROI (0.8 mm isotropic). As such, to utilize the constructed one, two or three-plane units for the LGN edge enhancement process, the 6 × 6 × 6 cube was downsampled to 3 × 3 × 3 cube with a 0.8 mm isotropic resolution to match the LGN containing image resolution. Examples of downsampled one, two, and three-plane units are presented in Figure 2.

The employed edge enhancement process was performed on a voxel-by-voxel basis over the entire LGN-containing ROI (extracted 22 × 22 × 22 cube) which consisted of 10,648 voxels. Each one of these voxels and their neighboring 3 × 3 × 3 voxels were fitted with one, two and three-plane downsampled units using the linear least squares regression (Larson et al., 2002) model shown in Eq. 1:


(1) I∼K⋅E+B

where *I* is defined as a 3 × 3 × 3 array formed by an image voxel and its immediate neighboring voxels, *E* is a 3 × 3 × 3 array representing the predefined downsampled units, *K* and *B* are the scalar fitting coefficients that are calculated to minimize the sum of the squared residuals (SSR) as depicted in Eq. 2.


(2) $$SSR=\sum_{i,j,k=1}^{3}{\left(I_{ijk}-K\cdot E_{ijk}-B\right)}^{2}$$

The *SSR* is minimized when its derivative with respect to the fitting *K* and *B* parameters reach zero, shown below by Eqs 3, 4:


(3) $$\frac{\partial SSR}{\partial K}=0$$

(4) $$\frac{\partial SSR}{\partial B}=0$$

Substituting the *SSR* from Eq. 2 into Eqs 3, 4, values for the fitting parameters of *K* and *B* were then calculated from the Eqs 5, 6 below:


(5) $$K=\frac{\sum_{i,j,k=1}^{3}E_{ijk}\cdot \left(I_{ijk}-\left\langle I\right\rangle \right)}{\sum_{i,j,k=1}^{3}E_{ijk}\cdot \left(E_{ijk}-\left\langle E\right\rangle \right)}$$

(6) B=⟨I⟩-K⋅⟨E⟩

**FIGURE 3:**
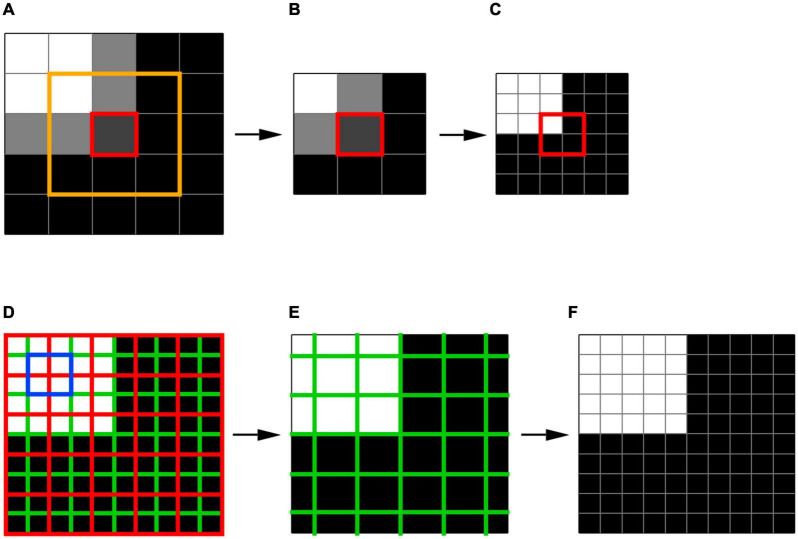
Voxelwise edge-enhancement process. **(A)** An example image containing 5 × 5 × 5 voxels. The red box denotes a single voxel and its immediate 3 × 3 × 3 neighboring voxels shown by the orange box. **(B)** The best fit two-plane edge 3 × 3 × 3 voxel unit for the fragment of the image in panel **(A)** enclosed in the orange box. **(C)** The two-plane edge 6 × 6 × 6 voxel unit corresponding to the best fit 3 × 3 × 3 voxel unit shown in panel **(B)**. The red box contains 2 × 2 × 2 voxels that represent the high-resolution version of the low-resolution voxel shown in red box in panel **(A)**. **(D)** The high-resolution version of the image shown in panel **(A)** resulted from the substitution of the low-resolution voxels by their high-resolution counterparts as shown in panels **(A–C)**. Each cell of the red grid contains 2 × 2 × 2 voxels taken from the centers of the best fit 6 × 6 × 6 voxel unit as shown in panel **(C)**. The signal averaging was performed in voxels constructing the green grid (example shown in blue box) to reduce the image noise (noise is not depicted). **(E)** The low-resolution image resulted from the signal averaging inside the cells of green grid. **(F)** The edge-enhanced high-resolution image obtained from the low-resolution image **(E)** by voxelwise edge-detection process shown in panels **(A–C)**. Thus, signal averaging step shown here reduces noise while preserving the integrity of the image, which is evident as an identity of images shown in panels **(D,F)**.

Thus, by setting the derivatives of *SSR* to zero, for each unit *E* (downsampled planes) corresponding fitting parameters for *K* and *B* are calculated. Then, the best fitted downsampled unit *E* is identified according to the minimum *SSR* (Eq. 2). We chose the minimum *SSR* as a criterion for the best fit and selection of the unit *E* representing the local feature of the image. The method of least squares is a standard approach in regression analysis to find the best approximation by minimizing the sum of squared deviations from the mean value. Using other criteria, e.g., the minimum of the sum of the absolute deviations from the median, doesn’t provide an analytical solution, and oftentimes leads to an ambiguous results. For example, for a selected center voxel (red box) and its three neighboring voxels (yellow box) in a fragment of an image (5 × 5 voxel box) shown in Figure 3A, the best fit was a two-plane unit that is shown in Figure 3B. The acquired *K* and *B* parameters for this plane is then applied to the corresponding 6 × 6 × 6 high resolution voxel unit which is shown in Figure 3C to acquire the best fitted high resolution plane as depicted in the red box of Figure 3C. It is important to note that the final edge enhanced processed voxel shown in Figure 3C (red box) now contains a 2 × 2 × 2 voxel cube which is twice the original native resolution (red box in Figure 3A).

To further enhance the visibility of the extracted edge, we applied voxelwise signal intensity averaging with resampling to reduce the image noise without affecting the edge location. An example of the averaging process is shown in Figures 3D–F. The box shown in Figure 3D depicts a fragment of an image (5 × 5 × 5 voxel box) that has already undergone edge detection process, resulting in a fitted two-plane unit with high resolution 0.4 mm isotropic voxels. Signal intensity was then averaged and substituted for each of the green voxels to construct a new representation of Figure 3D that contained new averaged signal intensity values (Figure 3E). Subsequent to signal averaging, the image underwent the edge-enhancement with upsampling (shown in Figure 3F). While the new resampled image, shown in Figure 3F, preserves the fitted planes and resolution created by the edge detection process, it contains averaged signal intensity in each voxel that dramatically improves image signal by reducing the voxel noise level. This two-step process of edge detection and signal averaging was repeated multiple times (e.g., ten times) in order to obtain an optimal edge with the least amount of image distortion and highest visibility for reliable LGN delineation. Our final iterative process, however, showed that three time repetition of the two-step process generated optimal edge enhanced LGN images.

### Application of the Edge Enhancement Algorithm to 3D MPRAGE Images

**FIGURE 4:**
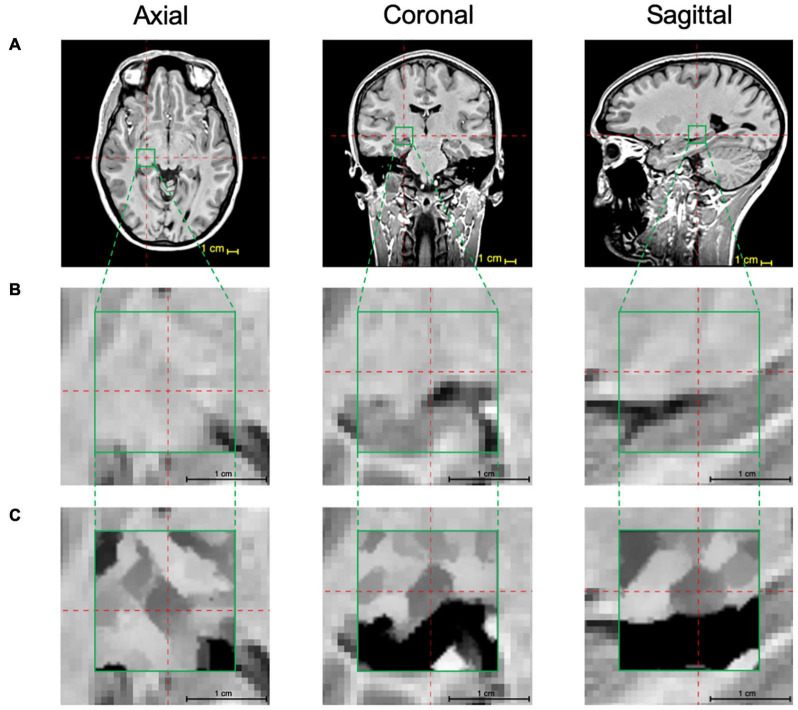
LGN edge enhancement process: **(A)** Using the 3D MPRAGE T1 weighted images the center coordinates of the LGN are identified on the axial, coronal, and sagittal scans (red crosshairs) and a 22 × 22 × 22 voxel ROI (green box) containing the LGN in its entirety is extracted. **(B)** The zoomed presentation of the LGN containing extracted ROI in the native space. **(C)** The edge enhanced ROI using the 3D-edge enhancement algorithm. For an improved visibility, the image inside the green box underwent contrast adjustment around intensity at the image center. Comparison of panels **(B,C)** clearly depicts the superior visibility of the LGN and the feasibility and immense advantage of the edge enhancement algorithm in improving LGN conspicuousness.

The above-mentioned two-step iterative process of the edge detection algorithm was pictorially depicted for simulated image fragments on a voxel-by-voxel bases. This process was then applied to the MPRAGE T1 images to improve LGN visibility. As mentioned earlier, to shorten the processing time, the edge enhancement algorithm was applied only to a small segment (ROI) of the whole brain MPRAGE scan that contained the LGN. This step was performed by locating the center of the LGN on axial, coronal, and sagittal images as depicted in the top row of Figure 4 (LGN containing ROI is shown in the green box). A zoomed representation of the T1 weighted image containing the extracted ROI in all three orientations (green box), is shown in the middle panel of Figure 4. The zoomed images clearly depict that the LGN is barely visible and difficult to delineate. The procedure is described in detail in the Supplementary Information section. Each voxel of this LGN-containing ROI (green box) then underwent the edge-enhancement procedure following Eqs 1–6. An example of the edge-enhanced LGN-containing ROI is shown in the bottom row of Figure 4. The comparison between the middle and bottom row clearly shows the increased visibility of the LGN and the efficacy of the edge enhancement algorithm employed that considerably increases the reliability of LGN delineation.

## Results

To validate the accuracy of the proposed algorithm in determining LGN volume while enhancing its visibility, we used a known geometric shape such as a cube and simulated it in an environment with various amounts of noise. The validation was designed to assess: (i) whether the algorithm of the edge-enhancement counteracts partial volume effects; (ii) the accuracy of volumetric measurements at various image noise levels; (iii) the accuracy of a delineated shape at various noise levels; (iv) whether the algorithm of the edge-enhancement distorts the shape of an object.

The cube shape was chosen since it can be represented as a set of 1-plane (cube’s faces), 2-plane (cube’s edges), and 3-plane (cube’s vertices) units as defined in the section “Methods” (see Figure 2). The cube dimension was set to 5.6 mm (7 voxels) to get a volume of 175.6 mm^3^ lying in the midrange of the human LGN volume (Bürgel et al., 1999). To evaluate how well the algorithm offsets the partial volume effects, we chose the setting where all faces of a cube contained partial volume voxels (see Figure 1B). The cube contrast was set to 1. The additive Gaussian noise (Hansen and Kellman, 2015) was used to introduce various levels of noise. The standard deviation of the Gaussian noise (σ) was set to 1/16, 1/8, 1/4, 1/2, or 1 which corresponded to the contrast to noise ratio (CNR) of 16, 8, 4, 2, or 1, respectively. Dice similarity coefficient (DSC) was used to assess the accuracy for shape delineation:


(7) $$DSC=\frac{2\cdot V_{shared}}{V_{cube}+V_{delineation}}$$

where *V*_*shared*_ is the shared volume between the cube and delineated edge-enhanced image, *V*_*cube*_ is the volume of the cube, *V*_*delineation*_ is the edge-enhanced cube delineated volume.

**FIGURE 5:**
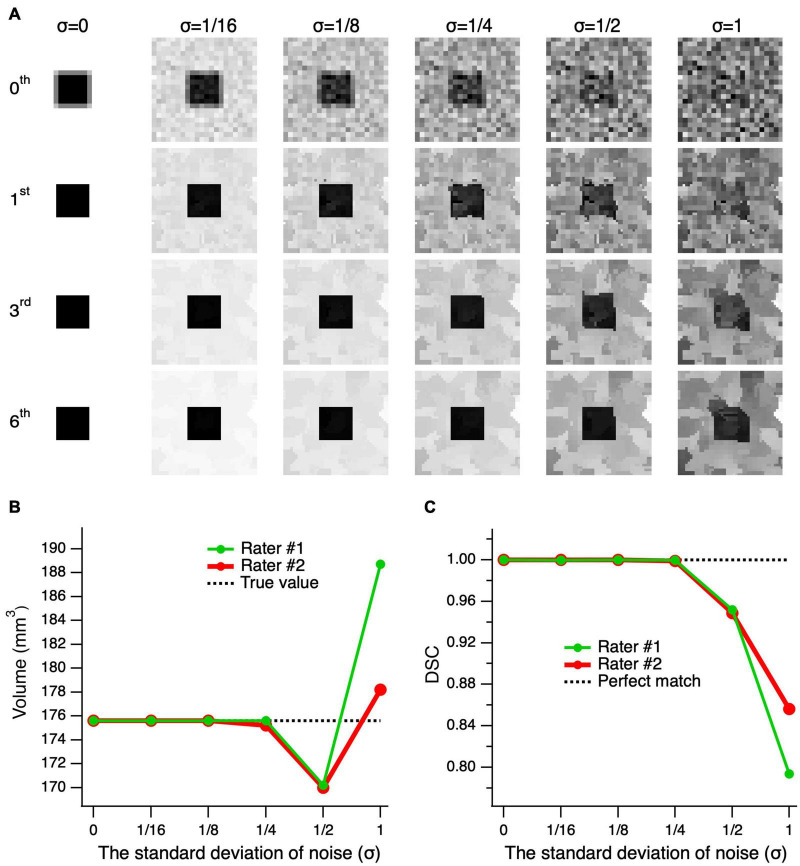
Performance of the edge-enhancement method at various image noise levels. **(A)** A cube’s central slice image with contrast of 1 and edge enhanced images at 0, 1, 3, and 6 iterations (top-bottom) of the cube’s center image with added noise levels of 0–1 standard deviation (σ) (left-right). The zeroth-order iteration corresponds to the cube’s unprocessed images at various noise levels (top row). Note that the higher the image noise the larger number of iterations needed to arrive at an image quality suitable for delineation. **(B)** Comparison of the volume measures of a simulated cube, post-processed at six consecutive edge enhancement iterations, between two raters and as compared to the ground truth (actual cube volume) at various noise levels. **(C)** The Dice similarity coefficient (DCS) between the true shape of the cube and delineations made by rater #1 (green) and rater #2 (red) on the images with various noise levels.

Figure 5 shows the results for edge-enhanced cube images with various noise levels and 0–6 iterations. The top row, 0th order iteration, represents the unprocessed noisy images of the cube with partial volume at various noise levels (left-right). As depicted in the second row, subsequent to the application of only one edge enhancement iteration, at lower noise levels (σ ≤ 1/8), the partial volume effects were resolved. To attain an image quality that is sufficient for a reliable delineation, particularly at higher noise levels, up to six consecutive edge-enhancement iterations are required, as seen for the bottom row images of Figure 5A (σ ≥ 1/4). The mean volume of the delineations made by the two raters was almost identical to the actual cube volume of 175.6 mm^3^ at noise levels σ ≤ 1/4, and differed by 5.5 ± 0.1 mm^3^ (3.1%) and 7.9 ± 7.4 mm^3^ (4.5%) at σ = 1/2 and 1, respectively. The mean DSC, which assesses the degree of shape matching between the true cube shape and the delineated shapes, showed a 100% matching at σ ≤ 1/4, 95.0 ± 0.2% at σ = 1/2, and 82.5 ± 4.4% at σ = 1. Thus, assuming an acceptable error of 5%, the edge-enhancement algorithm provided an accurate volumetric measurement at noise levels σ ≤ 1, and accurate delineation of a shape at noise levels σ ≤ 1/2.

**FIGURE 6:**
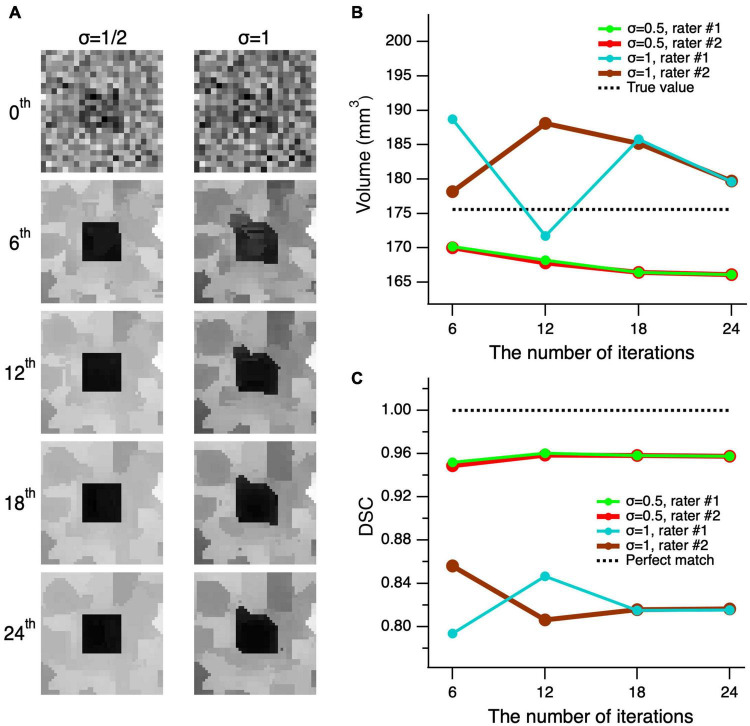
Convergence of the edge-enhancement method at high image noise levels. **(A)** A cube’s center slice image with contrast of 1 and edge enhanced images at various iterations (top-bottom) and added noise levels with standard deviation (σ) of 1/2 and 1 (left-right). The 0th order iteration corresponds to the unprocessed images of the cube’s central slice (top row). Note that the quality of the processed image at six iterations is acceptable for a reliable delineation. Although, using additional iterations incrementally improves image quality, the increase in processing time outweighs the moderate increase in image quality. **(B)** Comparison of the volume measures of a simulated cube, post-processed at various edge enhancements between 0 and 24 iterations at two different noise levels of 1 and 0.5. **(C)** The Dice similarity coefficient (DCS) between the true shape of the cube and delineations made by rater #1 (green) and rater #2 (red) on the images with σ = 1/2 and 1 that were processed with 6–24 consecutive edge-enhancements.

In order to test whether the edge-enhancing algorithm distorts the shape of an object, we performed a number of consecutive edge-enhancements iterations. We assumed if the algorithm indeed distorts the shape of an object, the distortion would be accumulated with each additional iteration and the object distortion would be revealed at the large number of edge-enhanced iterations. If the algorithm does not distort the image, then the image along with its volume and DSC of the delineations, would converge. The testing was performed for the worst possible cases with the highest image noise levels of σ = 1/2 (left column) and 1 (right column) of Figure 6A. As depicted in Figure 6A, the top row shows the unprocessed noisy images of the cube and all processed images using 6, 12, 18, and 24 iterations are shown from top to bottom, respectively. As seen in the second row of Figure 6A, the image processed with six iterations is already of a good quality, and the additional iteration steps do not considerably add to the image quality, particularly considering the increase in computing time. The observations on the image quality are further supported by the quantitative volumetric and DSC measurements presented in Figures 6B,C. The mean volume of delineations performed by the two raters was rather independent of the number of iterations and differed from the true volume of 175.6 mm^3^ by 7.9 ± 1.6 mm^3^ (4.5%) at σ = 1/2, and by 6.5 ± 2.4 mm^3^ (3.7%) at σ = 1. The mean DSC between the true shape of the cube and the delineated processed shapes, made by the two raters, did not depend on the number of iterations, and differed from the unity (perfect match) by 4.37 ± 0.34% at σ = 1/2, and by 17.92 ± 0.50% at σ = 1. Thus, the proposed algorithm does not distort the shape, and both volume and shape converge at the large number of iterations.

The CNR of the real LGN in MRI images is impossible to measure due to the variability of the voxel intensities both within and the surrounding areas of the LGN tissue. However, we believe that the typical LGN MRI images used in the study qualitatively contained similar noise levels as the simulated cube images with σ = 0.5. In order to estimate the degree of improvement the edge-enhancement algorithm may contribute to the validity of an object’s volume, the two raters (MA and ML) performed delineation of the 0th order cube at σ = 0.5 noise level (shown on left in the top of Figure 6A) and with six number of edge enhancement iterations at the same noise level. The 0th order delineation volumes were 277.5 mm^3^ (rater #1) and 287.2 mm^3^ (rater #2), whereas the DSC for shape analyses were 0.746 (rater #1) and 0.752 (rater #2). The averaged volumes measured on unprocessed images, by both raters, were significantly larger than those measured, by the same raters, for the edge-enhanced cubes using six iterations (282.4 ± 6.9 vs. 170.1 ± 0.1 mm^3^, *p* = 0.028), whereas the corresponding DSCs were significantly lower (0.749 ± 0.004 vs. 0.950 ± 0.002, *p* = 0.015). These results demonstrate the application of the edge enhancement algorithm to images with high noise and partial volume uncertainty with significantly increasing the visibility while preserving the shape and volume of the object.

A second method of validation was also performed using the volumes of LGNs from a population-based template as the gold standard (see Supplementary Figure 1). The left and right LGN volumes were obtained using the template and the ITK-SNAP software (see Supplementary Figure 2) and the volumes were used as the gold standard (184.8 and 181.8 mm^3^ for the right and left LGN, respectively). To test the edge enhancement algorithm performance, synthetic LGN images were created by adding Gaussian noise to the population-based atlas and then improving the LGN contrast using the proposed edge enhancement method (see Supplementary Figure 3). The LGN volumes were then delineated using the post-processed synthetic images and the left and right LGN volumes measured to be 188 and 180.6 mm^3^, respectively. These volumes were within 2% of the LGN volumes measured directly on the template as the gold standards providing further proof for the validity of the proposed edge enhancement methodology (for more details see the Supplementary Information).

**FIGURE 7:**
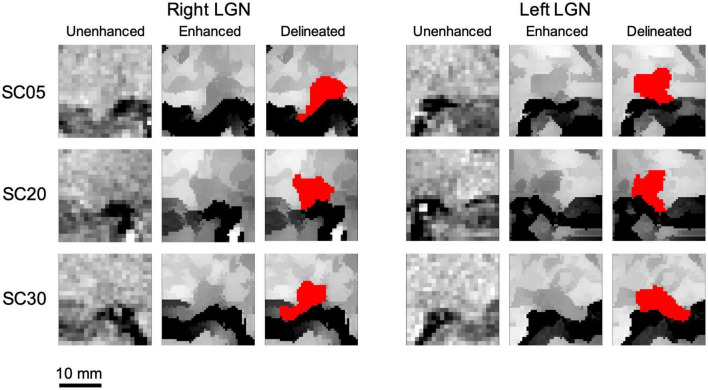
Examples of the unenhanced and edge enhanced lateral geniculate nucleus (LGN). As seen along the first column for both the left and right LGN, the LGN is hardly visualized for all unenhanced images. Subsequent to applying the edge enhancement algorithm, as seen along the second column of both the left and right LGN, the LGN can be clearly demarcated from the surrounding structures and can easily be manually outlined. An example of delineated left and right LGN (outlined in red) for three study participants are shown along the last columns for both left and right LGNs.

Subsequently, the edge enhancement algorithm was applied to the left and right LGN containing ROIs of all 19 participants. As demonstrated in Figure 4, instead of the whole brain, the enhancement process was performed only on a small extracted ROI containing the LGNs to shorten the processing time. As mentioned earlier, the resulted number of slices from a post-processed ROI was twice of those in the original acquisition (native space) with double in plane resolution (0.4 mm isotropic from 0.8 mm). Subsequent to the enhancement process, two raters (MA and ML) arrived at a consensus on demarcating the LGN borders visible on all MRI slices within the extracted ROI. Each rater then independently manually delineated the left and right LGN for all participants, using the ITK-SNAP software. Examples of the original unenhanced, edge enhanced, and delineated left and right LGNs for three participants are presented in Figure 7.

The dramatic improvement in visibility of the LGNs on edge enhanced images as compared to their corresponding unenhanced images is clearly demonstrated in Figure 7. The LGN volumes were calculated using the volume/statistic option of the ITK-SNAP segmentation menu. The mean and standard deviation of the right and left LGN measurements for both raters are presented in Table 2. The individual subject’s left and right LGN volume measures by both raters are presented in the Supplementary Table 1.

**TABLE 2:** Statistical evaluation of the inter-rater reliability for two raters on measurements of the left and right LGNs in 19 healthy participants.

	*N*	Rater 1 mean (SD) (mm^3^)	Rater 2 mean (SD) (mm^3^)	Difference mean (SD) (mm^3^)	95% Limits of agreement	Intraclass correlation (95% CI)
**Left LGN**	19	176 (7.9)	174 (8.1)	2.0 (5.4)	−8.6, 12.6	0.74
**Right LGN**	19	175 (8.9)	173 (8.9)	2.4 (5.0)	−7.4, 12.2	0.81

**FIGURE 8:**
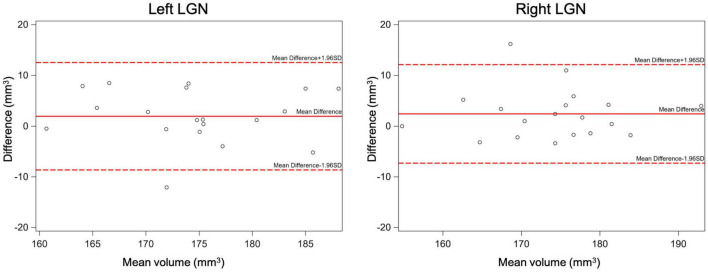
The inter-rater Bland-Altman plots for the left and right LGN volume measurements. As shown here the difference in the LGN volumes between the two raters for all subjects were mostly less than ±10 mm^3^.

The inter-rater reliability was assessed by calculating the mean difference, standard deviation (SD), and 95% limits of agreement (calculated as mean difference ±1.96*SD), supplemented with Bland-Altman plots (Figure 8). In addition, the intra-class correlation and its 95% confidence interval (95% CI) were calculated to assess agreement of measurements between the two raters. Results of the inter-rater reliability statistical assessment are presented in Table 2. As presented in Table 2, the intraclass correlations between the two raters for the left and right LGN were 0.74 and 0.81, respectively. The Bland-Altman plots shown in Figure 8 demonstrates the LGN volume differences between the two raters for the left and right LGN for all study participants. As shown in Figure 8, the difference in the LGN volumes between the two raters for all subjects were primarily ±10 mm^3^, except for the right LGN measurement of one participant.

**FIGURE 9:**
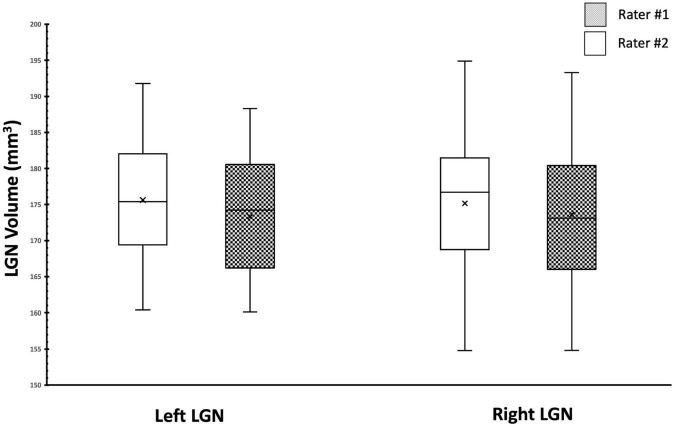
Box Whisker plot of the left and right LGNs for by both raters. Comparison of the center, spread of group and the median for the left and right LGN volumes shows great similarity between two raters.

We also assessed and compared the similarity of the measurements between the two raters using the Box and Whiskers plot (Figure 9). As shown in Figure 9, the center, spread of group, the median, and the whiskers that represent the ranges from the bottom 25% and the top 25% of the left and right LGN volumes measured by the two raters are very similar.

**FIGURE 10:**
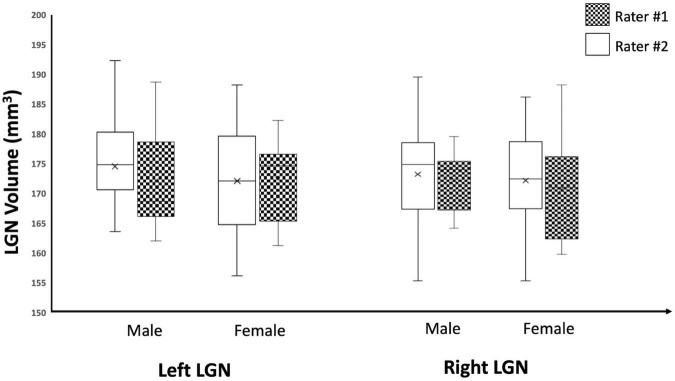
Box Whisker plot of the left and right LGNs for males and females reported by both raters. As depicted here, the left and right LGN volume measures reported by both raters did not show any sex differences.

As an additional test for the validity of the edge enhancement process, we also examined the effect of sex on the LGN volumes among study participants and, consistent with previous reports (Bürgel et al., 1999; Li et al., 2012; Kelly et al., 2014; Papadopoulou et al., 2019b), we found no significant differences in the volumes of the LGN between male and female participants. The study subjects consisted of 10 males and 9 females with mean ages of 16.2 and 12.1 years, respectively. The results for sex differences in LGN volume is presented in Table 3. Although the male population was slightly older than females, there were no significant differences between the two populations (*p* = 0.27). There were no significant differences for the left and right LGNs volume measures reported by both raters (*p*-values 0.59–0.81), as shown in Table 3 and the Box Whisker plot in Figure 10.

**TABLE 3:** Sex differences in LGN volume measured in 10 males and 9 females.

Subject gender (*n*)	Rater 1		Rater 2		Age (years)
	Left LGN mean ± SD (mm^3^)	Right LGN mean ± SD (mm^3^)	Left LGN me an ± SD (mm^3^)	Right LGN mean ± SD (mm^3^)	
Male (10)	176.59 ± 9.01	175.76 ± 11.89	174.49 ± 9.10	174.54 ± 10.71	16.2 ± 9.9
Female (9)	174.58 ± 7.49	174.72 ± 5.85	172.789 ± 7.73	172.81 ± 10.46	12.11 ± 4.62
Two-tailed *p*-value	0.59	0.81	0.66	0.72	0.27

## Discussion

The lateral geniculate nucleus (LGN) is a small multilayered and inhomogeneous structure that receives major sensory inputs from the retina and plays a critical role in the transfer of visual information to the visual cortex. Although there has been great interest in the morphometry of the LGN, with respect to various retinal conditions, currently available methods have not met the complexity and challenges involved in LGN imagery. Using a routine T1-weighted 3D-MPRAGE imaging sequence acquired on a 3T MRI system, we performed a morphometric evaluation of the LGN on a group of normal subjects by overcoming image noise and volume uncertainty caused by the partial volume artifacts. The increase in contrast and signal to noise ratio of the LGN containing images resulted in a higher reliability and accuracy for LGN delineation. This improvement in LGN visibility was possible through the development of a post-processing algorithm utilizing a novel method of edge enhancement with upsampling. The method is based on modeling a small brain structure, such as the LGN, as a polyhedron with faces, edges, and vertices fitted with a local plane, the intersection of two planes, and the intersection of three planes, respectively. Representation of 3D-biomedical shapes as polyhedrons has shown to be accurate and allows for precise analytical calculations in closed form Fourier transform expressions (Ngo et al., 2016). Any polyhedron’s face or edge can be accurately fitted with one-two-plane units (such as shown in Figure 2), respectively. However, the number of crossing planes that form a vertex can vary from three (e.g., vertices in a cube and triangular pyramid) to four (e.g., apex of a square pyramid), or even more. Here, to reduce the computational time, we limited the number of planes crossing at a vertex to three. A polyhedron with such limited numbers of plane crossings (three planes) to form vertices would accurately represent many 3D- shapes, such as a cube or triangular-based pyramid. Application of this post-processing algorithm on the native images acquired on a typical MRI system (3T Magnet) using a routine clinical protocol (e.g., 3D T1 weighted) dramatically improved the visualization of the LGN (see Figures 4, 7). To achieve a similar contrast, previous studies have employed MR sequences not routinely used in a clinical setting, which required over one hour of acquisition time (Kelly et al., 2014; McKetton et al., 2014, 2015; Moro et al., 2015; Grigorian et al., 2016; Giraldo-Chica and Schneider, 2018; DeSimone and Schneider, 2019), focusing only on improving LGN visualization, as compared to a short 7-min sequence used as part of a routine clinical protocol (e.g., 3D MPRAGE).

The validation of the method demonstrated that the algorithm of the edge-enhancement does counteract partial volume effects while not distorting an object’s shape at contrast to noise ratio as low as 2 (see Figures 5, 6). Though the image quality was good enough for delineating after six edge-enhancement iterations, the delineations were more consistent between raters when the number of iterations reached 18–24 (Figures 6B,C). Here we demonstrate the performance of the edge enhancement algorithm on a small structure, on the order of the LGN volume, with similar noise levels and show increased object visibility. Thus, we expect similar performance for the algorithm on the real LGN-containing MRI images that would enhance the visibility of LGN increase its morphometric accuracy. It is worth noting that the image of the test cube with added noise may not mimic the image quality of the LGN dataset, as the real LGN images consist of multiple objects with various contrasts (inhomogeneous object). The selected test cube image was used to demonstrate that the proposed algorithm counteracts the partial volume effects, increase object visibility at typical MRI image noise level, and preserves the shape of an object to provide an accurate volumetric measurement for typical MRI images with low contrast and high noise levels.

The LGN volume measurements in our study were highly consistent between the raters, with the inter class correlation coefficients (ICCs) of 0.74 and 0.81, which are above the minimum value of ICC of 0.70 for reliable measurements (Cohen, 2001). As mentioned earlier, such high reproducibility rates among two raters for the LGN volume measurements from a low contrast and noisy 3D images, as part of routine imaging protocol, is comparable to images acquired on a 3T systems with multiple signal averaging of up to 30–40 using a proton density weighted sequence with a long acquisition time (Kelly et al., 2014; McKetton et al., 2014, 2015; Moro et al., 2015; Grigorian et al., 2016; Giraldo-Chica and Schneider, 2018; DeSimone and Schneider, 2019). However, the LGN volumes are hardly consistent between the previously reported studies (Table 4), ranging from 76 mm^3^ (Li et al., 2012) to 267 mm^3^ (Korsholm et al., 2007), with the average of the left and right LGN volumes of 149.6 ± 45.8 mm^3^ (mean ± SD, *n* = 27). While the imaging protocol, particular sequence parameters, strength of the magnetic fields, head coils used, and the delineation methodology play a major role in the outcome of this discrepancy, one can hypothesize that such a wide range between the low and high values reported for the LGN volumes might be primarily caused by volume uncertainty due to partial volume artifacts. As a result, the LGN volume is underestimated in some studies and overestimated in others. Interestingly, the LGN volume reported using a 7T system is substantially lower in volume as compared to the volumes reported by others and those presented in the current study. This discrepancy is not caused by an overestimation by the proposed algorithm and is clearly depicted in the validity section where a known cube volume is accurately assessed by the algorithm at various noise levels. Moreover, as shown in Table 4, the LGN volumes measured on the 7T systems were consistently and considerably lower than those measured using 3T MRI units (see Table 4, except for Giraldo-Chica and Schneider, 2018). The reason for this discrepancy is not clear, but one factor could be the iron content of the LGN. As reported by Müller-Axt et al. (2020), the magno- and parvo- cell layers of LGN both contain ferric ion and minute amount of any ferromagnetic substance would cause reduction in MR signal intensity and this effect is much higher at higher magnetic fields. Also, according to Ramos et al. (2014), there is an age-related increase in brain iron concentration levels and this could play an important role in calculating the LGN volume as well. It is important to note that the average age of our subjects was 14 years, and the average age of subjects reported by Schmidt et al. (2018) was 47 years. Another factor could be due to large variations in LGN volumes among subjects, even up to twofold as reported in a post-mortem study (Andrews et al., 1997).

**TABLE 4:** Previously reported LGN volumes.

Left LGN (mm^3^)	Right LGN (mm^3^)	Average (mm^3^)	Method	References
76.5 ± 14.3	86.2 ± 11.1	81.3 ± 12.7	1.5T Signa, GE, T1-weighted, 3D fast-spoiled gradient sequence, 1 × 1 × 1 mm^3^, *in vivo*, automatic segmentation.	Li et al., 2012
87.7 ± 10	88.8 ± 11	88.3 ± 10	7T Magnetom Terra, Siemens, 3D MP2RAGE, 0.8 × 0.8 × 0.8 mm^3^, *in vivo*, manual segmentation.	Schmidt et al., 2018
NA	95.9 ± 13.5	NA	Nissl-stained brain sections, manual point counting.	Gupta et al., 2006
92.7 ± 24.4	106.1 ± 24.3	99.4 ± 24.4	7T Magnetom, Siemens, *in vivo* PD-weighted imaging, manual segmentation.	Lee et al., 2014
116 ± 18	100 ± 26	108 ± 22	1.5T Magnetom, Siemens, *in vivo*, T1-weighted 1 mm isotropic MPRAGE, 1 × 1 × 1 mm^3^, semi-automatic segmentation.	Renauld et al., 2016
120.7 ± 6.2	112.3 ± 7.0	116.5 ± 6.6	3T MRI scanner, proton density images, manual segmentation.	Giraldo-Chica and Schneider, 2018
113.5 ± 13.3	120.9 ± 14.0	117.2 ± 13.7	7T Magnetom, Siemens, 3D- MP2RAGE, 0.5 × 0.5 × 0.5 mm^3^, *in vivo*, manual segmentation.	Müller-Axt et al., 2020
115	121	118	Nissl-stained brain sections, manual point counting.	Andrews et al., 1997
119 ± 22	NA	NA	7T Magnetom, Siemens, 3D- MP2RAGE, 0.7 × 0.7 × 0.7 mm^3^, *in vivo*, manual segmentation.	Müller-Axt et al., 2017
127.6 ± 32.0	111.9 ± 26.1	119.8 ± 29.1	T1-weighted MRI scans, automatic segmentation.	Wang et al., 2015
NA	NA	124 ± 21	7T, Philips, segmented MPRAGE, 0.4 × 0.4 × 0.4 mm^3^, *in vivo*, manual segmentation.	Aldusary et al., 2019
144.1 ± 32.6	116.8 ± 29.8	130.5 ± 31.4	3T, GE, T1-weighted, 1 × 1 × 1 mm^3^, *in vivo*, automatic segmentation.	Wang et al., 2016
143.1 ± 19.7	143.5 ± 22.3	143.3 ± 21.0	3T, Signa HDxt, GE, 3D BRAVO sequence, 1 × 1 × 1 mm^3^, *in vivo*, manual segmentation.	Dai et al., 2011
146.4 ± 18.4	145.2 ± 21.4	145.8 ± 19.9	3T, GE, 3D BRAVO sequence, 1 × 1 × 1 mm^3^, *in vivo*, manual segmentation.	Chen et al., 2013
147.0 ± 23.9	151.7 ± 15.7	149.4 ± 20.2	3T, Philips Intera, T1-weighted, 1 × 1 × 1 mm^3^, *in vivo*, automatic segmentation.	Hernowo et al., 2011
NA	NA	154.2 ± 16.5	1.5T, GE, 3D T1 SPGR sequence, 1 × 1 × 1 mm^3^, *in vivo*, manual segmentation.	Zhang Y.Q. et al., 2012
160 ± 18	157 ± 18	159 ± 18	3T Magnetom Trio, Siemens, PD-weighted, 0.8 × 0.8 × 0.8 mm^3^, *in vivo*, automatic segmentation.	Kelly et al., 2014
157.9 ± 9.8	165.2 ± 9.6	161.6 ± 9.7	3T Magnetom Trio, Siemens, PD-weighted, 0.75 × 0.75 × 0.75 mm^3^, *in vivo*, manual segmentation.	McKetton et al., 2014
145.5 ± 11.0	179.1 ± 15.8	162.3 ± 21.7	3T, Philips Ingenia, T1-weighted 3D-TFE, 1 × 1 × 1 mm^3^, *in vivo*, automatic segmentation.	Zhang et al., 2020
168.13	167.94	168	3T Magnetom Trio, Siemens, PD-weighted, 0.35 × 0.35 × 1 mm^3^, *in vivo*, manual segmentation.	McKetton et al., 2015
190 ± 37.7	167 ± 37.4	178.5 ± 38.4	Nissl stained brain sections, manual point counting.	Bürgel et al., 1999
NA	NA	185	Nissl stained brain sections, manual point counting.	Bush and Allman, 2004
NA	NA	191.4 ± 47.7	3T Prisma, Siemens, T1-weighted MPRAGE, 1 × 1 × 1 mm^3^, *in vivo*, manual segmentation.	Papadopoulou et al., 2019a
199 ± 37.5	188.2 ± 50.1	193.6 ± 43.4	3T Magnetom Trio, Siemens, PD-weighted, 0.75 × 0.75 × 1 mm^3^, *in vivo*, manual segmentation.	Grigorian et al., 2016
156.3 ± 20.6	240.3 ± 29.9	198.3 ± 49.4	3T Magnetom Trio, Siemens, T1-weighted MPRAGE, 1 × 1 × 1 mm^3^, *in vivo*, automatic segmentation.	Papadopoulou et al., 2019b
255 ± 14	251 ± 22	253 ± 18	3T Trio, Siemens, PD-weighted, 0.375 × 0.375 × 1 mm^3^, *in vivo*, manual segmentation.	DeSimone and Schneider, 2019
NA	NA	267 ± 27	3T Magnetom Trio, Siemens, T1-weighted MPRAGE, 1 × 1 × 1 mm^3^, *in vivo*, manual segmentation.	Korsholm et al., 2007

*The volumes (mean ± SD mm^3^) are shown for left LGN (1st column), right LGN (2nd column), and average of left and right LGNs (3rd column).*

Whereas the measurements of the absolute volume of the LGN are subject to volume underestimations or overestimations due to the partial volume effect, the relative volumes of the LGN in the right and left hemispheres and in male vs. female brains should not be. Therefore, we expected higher consistency between studies on comparisons of the LGN laterality, and gender dependence across studies as well as what is presented here. Based on our results, we observed a lack of laterality in LGN volume measures (left LGN volume = 175 ± 8 mm^3^; right LGN volume = 174 ± 9 mm^3^), which was consistent with some reports (Dai et al., 2011; Chen et al., 2013; Kelly et al., 2014; McKetton et al., 2014, 2015; Grigorian et al., 2016; Giraldo-Chica and Schneider, 2018; Schmidt et al., 2018; DeSimone and Schneider, 2019). However, our results did not corroborate studies reporting asymmetric LGN volume measures, with reported left larger LGN (Bürgel et al., 1999; Lee et al., 2014; Wang et al., 2015, 2016; Renauld et al., 2016), or right larger LGN (Andrews et al., 1997; Li et al., 2012; Papadopoulou et al., 2019a,b; Müller-Axt et al., 2020; Zhang et al., 2020). Furthermore, we observed no LGN volume differences between our male and female participants which again is consistent with most previous reports (Bürgel et al., 1999; Li et al., 2012; Kelly et al., 2014; Papadopoulou et al., 2019b). However, there are two studies that report a larger LGN volumes for males as opposed to females (Wang et al., 2015; Papadopoulou et al., 2019a). The reason for the observed inconsistency for laterality and gender differences in LGN volumes among previous reports remains unclear. Although, we believe the scanning, clarity, visibility and delineation methodology may play an important role for all studies targeting LGN morphometry. Previous studies have established a 15% reduction in structural volume between the ages of 20 and 70 years (Li et al., 2012). And histologic analysis of post-mortem tissue assessing the LGN volumes report an even higher percentage (30%) of reduction in volume by age (Selemon and Begovic, 2007). It is important to point out that our study participants, with a median age of 11 and mean age 14, were much younger than previously reported populations in studies concerning the LGN volume measurements.

An important point regarding the current edge detection algorithm is that the proposed method is not limited to the enhancement and delineation of the LGN structures only. Indeed, a modified version of the current program with an expanded initial ROI size can be used to enhance and improve the visibility of all deep gray matter structures such as caudate, putamen, amygdala, hippocampus, internal/external capsule, substantia nigra, and claustrum, to name a few, that are susceptible to noise and partial volume artifacts. The usefulness of the proposed algorithm in improving the contrast to noise of a segment of claustrum (challenging structure to extract) in a typical subject is presented in Supplementary Figure 4. This example clearly demonstrates the applicability of the proposed edge enhancement algorithm to other small gray matter structures.

The major shortcoming of the proposed method is a rather long computational time (∼4 h/image on 4 GHz Quad-Core Intel Core i7 with single-thread processing), though the post-processing time occurs outside the imaging time and does not affect patient care. Our initial attempt to shorten the processing time was to restrict the number of predefined units by limiting the maximum number of planes forming a vertex to only three planes. However, this limitation would result in a less accurate representation for the vertices that are composed of larger number of crossing planes, such as the apex of the rectangular-based pyramid that is formed by four planes. To reduce processing times, our future goal is to develop an algorithm that fits fewer parameters to a 3D- multiplane edge unit rather than finding the best fit out of thousands of predefined units. The computation time considerably reduces when using an analytical approach and allows modeling of polyhedron vertices with up to four crossing planes (instead of three) with an unlimited spatial resolution (instead of only twofold). While reducing the computation time, the proposed methodology has the potential to further decrease the partial volume uncertainty, increase the accuracy of the delineations, and potentially allow for automatic segmentation of the structure of interest.

## Data Availability Statement

The original contributions presented in the study are included in the article/Supplementary Material, further inquiries can be directed to the corresponding author.

## Ethics Statement

The studies involving human participants were reviewed and approved by IRBs at University of Pennsylvania and Children’s Hospital of Philadelphia. Written informed consent to participate in this study was provided by the participants’ legal guardian/next of kin.

## Author Contributions

ML and MA conceived, designed, and implemented the idea, and wrote the manuscript. MA and JB organized and performed the data acquisition. G-SY and YY performed statistical analysis of the data and interpretation of the results. All authors contributed to reviewing the manuscript.

## Conflict of Interest

The authors declare that the research was conducted in the absence of any commercial or financial relationships that could be construed as a potential conflict of interest.

## Publisher’s Note

All claims expressed in this article are solely those of the authors and do not necessarily represent those of their affiliated organizations, or those of the publisher, the editors and the reviewers. Any product that may be evaluated in this article, or claim that may be made by its manufacturer, is not guaranteed or endorsed by the publisher.

## Footnotes

1
http://www.itksnap.org

2
www.wavemetrics.com
