# Supplementary material for: Improving the Quantification of the Lateral Geniculate Nucleus in Magnetic Resonance Imaging Using a Novel 3D-Edge Enhancement Technique
Source: Front Comput Neurosci. 2021 Dec 3;15:708866. doi: 10.3389/fncom.2021.708866 (PMC8677828; doi:10.3389/fncom.2021.708866)
Supplement: Supplementary file 1 [file Data_Sheet_1.docx]

|  | Rater 1 | | Rater 2 | |
| --- | --- | --- | --- | --- |
| Subject | Left LGN (mm)^3^ | Right LGN (mm)^3^ | Left LGN (mm)^3^ | Right LGN (mm)^3^ |
| SC05 | 184.5 | 177.7 | 181.6 | 173.6 |
| SC07 | 160.4 | 154.8 | 160.9 | 154.8 |
| SC13 | 170.8 | 178.6 | 162.3 | 193.3 |
| SC14 | 167.2 | 163.1 | 163.6 | 166.3 |
| SC16 | 165.9 | 165.2 | 178 | 160 |
| SC17 | 168 | 169.1 | 160.1 | 165.7 |
| SC18 | 175.6 | 178.1 | 175.2 | 179.5 |
| SC19 | 174.5 | 176.7 | 175.6 | 160.5 |
| SC20 | 171.6 | 168.4 | 172.2 | 170.6 |
| SC28 | 178.2 | 181.2 | 169.8 | 170.2 |
| SC30 | 175.4 | 170.9 | 174.2 | 169.9 |
| SC02 | 176 | 179.6 | 174.7 | 173.7 |
| SC06 | 175.2 | 172.6 | 179.2 | 176 |
| SC08 | 171.6 | 175.5 | 168.8 | 173.1 |
| SC09 | 181 | 181.7 | 179.8 | 181.3 |
| SC26 | 183.1 | 183 | 188.3 | 184.8 |
| SC29 | 191.8 | 194.9 | 184.4 | 190.9 |
| SC31 | 177.6 | 175.8 | 170 | 177.5 |
| SC32 | 188.7 | 183.2 | 181.3 | 179 |

**Table S1.** Detailed volume measurements of the left and right LGN for the individual study participants.

**Algorithm Validation Using Population-Based Atlas**

Here, we further verify the validity of the proposed edge detection algorithm on a population-based atlas constructed from all 19 study participants using the ANTs software (<http://stnava.github.io/ANTs/>). The red boxes in Figure S1 presents all the images where the left and right LGN sections were visible on the atlas.


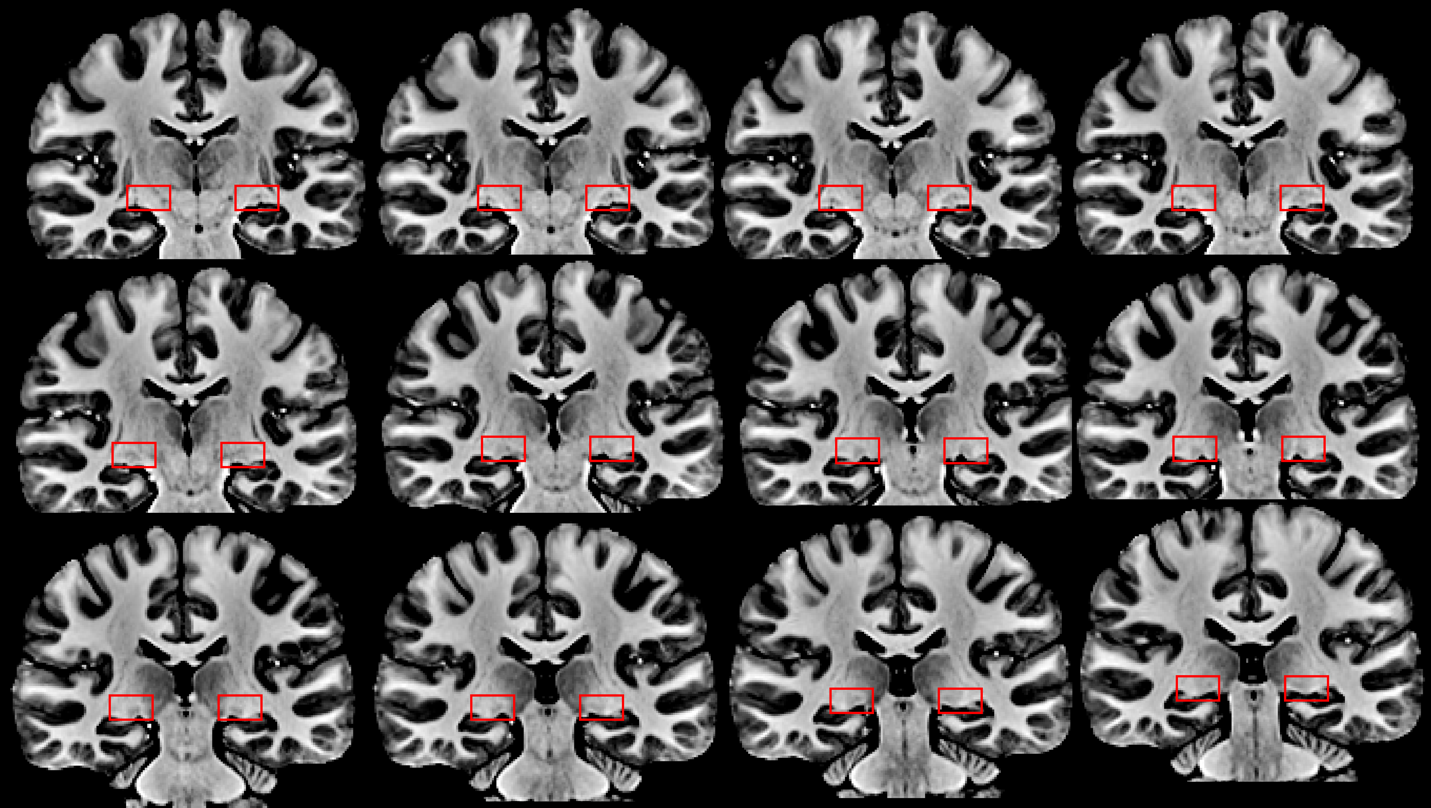


**Figure S1.** Identifying LGN cross sections on a population-based atlas built using ANTs (<http://stnava.github.io/ANTs/>) with 0.8 mm isotropic resolution.

The left and right LGNs were then delineated using the ITK-SNAP software and the identified LGN cross sections on the population-based atlas (see Figure S2).


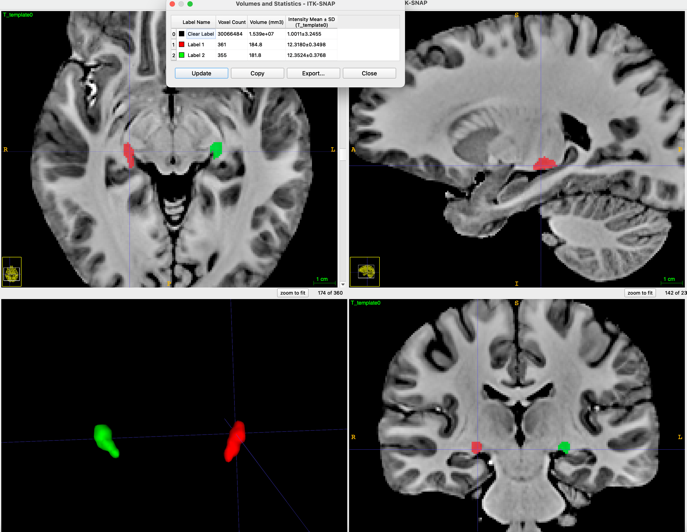


**Figure S2**. The left and right LGN delineations using a population-based template resulted in LGN volumes of 184.8 and 181.8 mm^3^ for the right and left LGN respectively.

Although the ANTs registration algorithm is proven to be one of the best methods for the brain MRI images, it is still suboptimal for the registration of small gray matter structures such as LGN. In fact, the recurring mis-registration between cases for the small gray matter structures would become accumulative and spread around the edge of the structure, resulting in a larger volume than the original structure. As expected, the population-based template produced larger LGN volumes of 184.8 and 181.8 mm^3^ for the right and left LGN respectively as compared to the average volume calculated on individual study participants.

To test the edge enhancement algorithm performance, synthetic LGN images needed to be created using the population-based atlas and then improved using the proposed enhancement method. First, the center coordinates of the left and right LGNs were identified on the population template and a 22x22x22 voxels ROI containing the LGNs in their entirety were extracted from the whole brain (Figure S3, column A) atlas images. Gaussian noise was then added to the images to synthetically create low contrast/high noise images of the left and right LGNs (Figure S3, column B); the standard deviation of the noise was the half of calculated noise on the template image. The images with the added noise were then processed using the proposed edge enhancement algorithm (Figure S3, column C). Using the processed images, the left and the right LGNs were then delineated using the ITK-SNAP software (Figure S3, column D). The left and right LGN volumes were obtained from the processed synthetic images and measured to be 188 and 180.6 mm^3^ respectively. These volumes were within 2% of the LGN volumes measured directly on the template and used as the “gold standard”, providing additional proof for the validity of the proposed edge enhancement methodology.


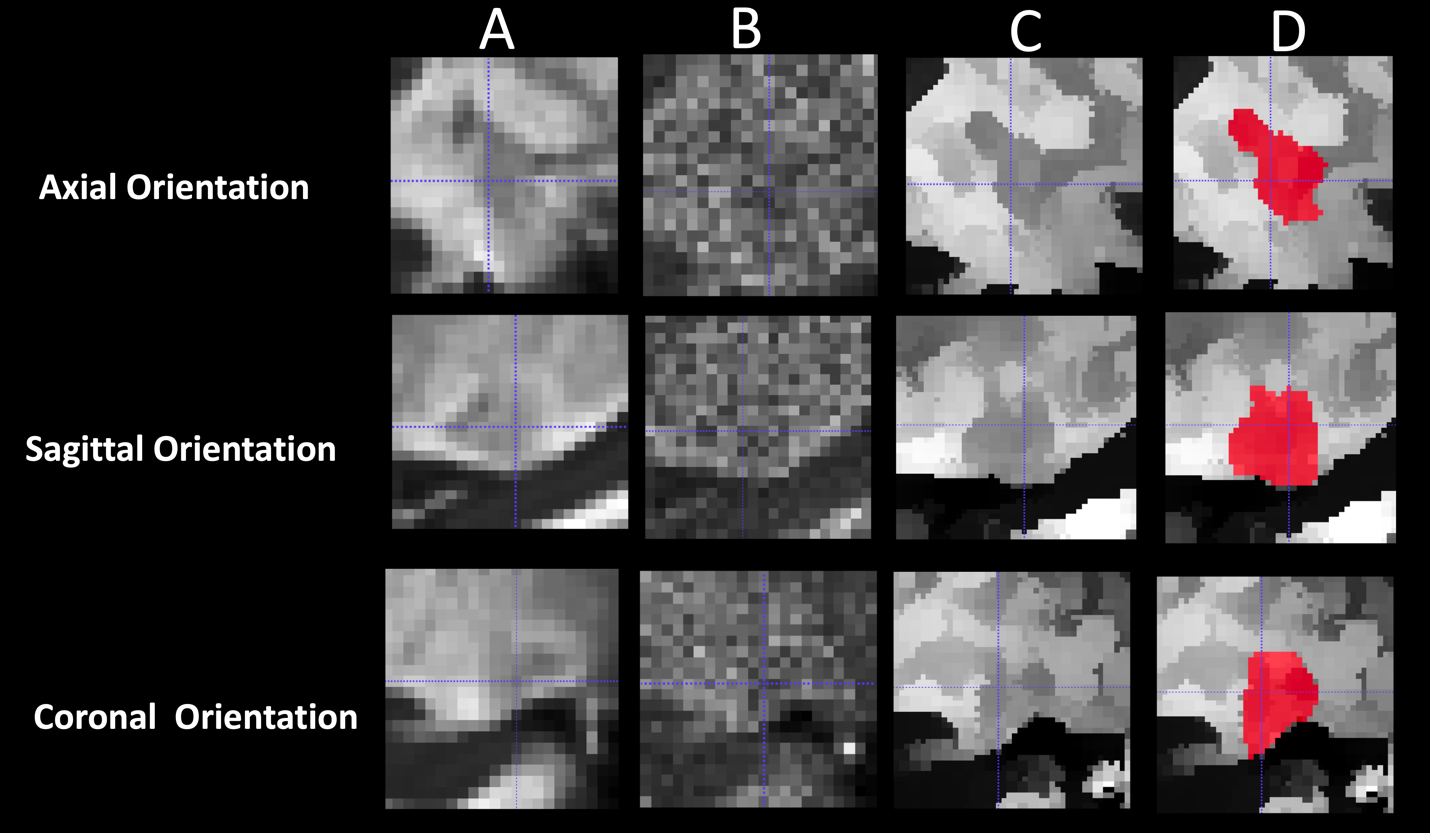


**Figure S3.** Processing and delineation of the right LGN using the population-based atlas. Column A shows an extracted 22x22x22 ROI from the atlas, containing the right LGN. The synthetic images were created by adding noise to the extracted ROI as shown in Column B. The edge enhanced processed right LGN images for all three orientations are presented in Column C. The visible right LGN sections were outlined using the ITK-SNAP software as depicted in red in Column D.

**Application of the Edge Enhancement Algorithm to Structures Other Than LGN**

An important point regarding the current edge detection algorithm is that the proposed method is not limited to the enhancement and delineation of the LGN structures only. Indeed, a modified version of the current program with an expanded initial ROI size can be used to enhance and improve the visibility of all deep gray matter structures such as caudate, putamen, amygdala, hippocampus, internal/external capsule, substantia nigra, and claustrum, to name a few, that are susceptible to noise and partial volume artifacts. The usefulness of the proposed algorithm in improving the contrast to noise of a segment of claustrum (challenging structure to extract) in a typical subject is presented in Figure S4. This example clearly demonstrates the applicability of the proposed edge enhancement algorithm for other deep gray matter structures.


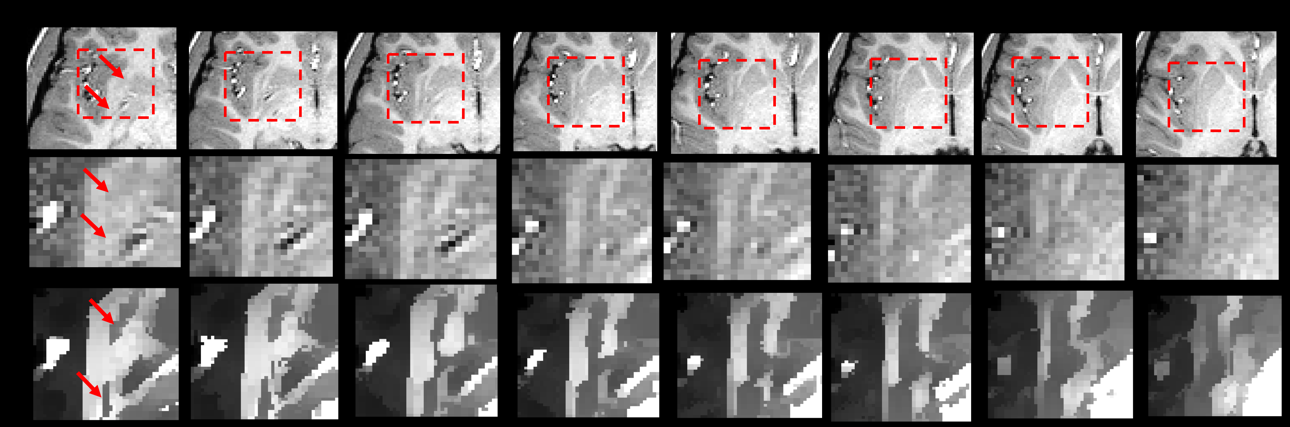


**Figure S4**. Improving visibility of claustrum. Serial (from right to left) MRI images of the claustrum-containing slices of a brain. *Top*: claustrum-containing regions are enclosed into the red dashed boxes. *Middle*: serial 22x22 voxels slices of unprocessed MRI image showing claustrum (pointed with red arrows). *Bottom*: processed images obtained from the raw images shown in the middle panel by applying the edge-enhancement algorithm. Note that the visibility of claustrum (pointed with red arrows) is significantly improved compared to that of the corresponding raw images shown in the middle panel.

**The Edge Enhancement Procedure**

The prerequisites for the image processing are the installed IgorPro and ITK-SNAP software. All the necessary files and documentation can be downloaded from GitHub repository https://github.com/Mikhail-Lipin/LGN.

The steps for using the edge enhancement procedure are the following:

1. Open command line terminal (macOS). Go to the folder with NIFTI MRI images. Set a path for c3d plugin in ITK-SNAP software (e.g., export PATH="/Applications/ITK-SNAP.app/Contents/bin/":$PATH). Copy shell script 3D_to_1D to the folder with NIFTI MRI images, and grant the shell script the executive privilege with command chmod ... .

2. Open MRI image in ITK-SNAP. Identify the center of LGN in a sagittal slice. Run the script 3D_to_1D with five arguments. The 1st, 2nd, and 3rd arguments are the x-, y-, and z-coordinates of the LGN center. The 4th argument is the name of the image file without extension ".nii.gz". The 5th argument is either R (right LGN), or L (left LGN). For example: ./3D_to_1D 120 120 120 MRimage R . The outputs of the shell script are the text file MRimage_R.txt and 22x22x22 excise of LGN containing image.

3. Open IgorPro experiment EdgeEnhancement.pxp in IgorPro. A permanent link for downloading IgorPro experiment EdgeEnhancement.pxp can also be found here: https://drive.google.com/drive/folders/1zTe98Gw6bCtnQUJpOLICDqUQEz1Rieec?usp=sharing . In IgorPro experiment menu Data => Load Waves => Load Delimited Text load file MRimage_R.txt and save it as MRimage_R

4. In IgorPro command line, run Ar2Im(MRimage_R). The output will be the 3D 22x22x22 image with the name of MRimage_R_Im. Type this name into the table ImName. The table ImName may contain as many 3D images as you want to run at once in the next step.

5. In IgorPro command line, run Batch(SL,Nmeans,Niterations), where the first argument SL is either 0 (Shorter processing time, preferred) or 1 (Longer processing time). The second argument Nmeans is 1 for shapes with vertices that can be represented by 3 crossing planes (e.g., cube or triangular pyramid), and 4 to 20 for shapes with vertices that cannot be represented by 3 crossing planes (e.g., sphere, or LGN). The third argument is the number of consecutive filtrations (6 for images with moderate noise level, 18 to 24 for images with high noise level). For example, to reproduce the image Cube_Im_1i in the root directory of IgorPro data browser, execute Batch(0,1,1) ("1i" at the end of the name Cube_Im_1i stands for "1 iteration"). The processing takes ~50 min/iteration/image.

6. When the previous step is finished, run Im2Ar(ImageName) in IgorPro command line, where ImageName is the name of the edge-enhanced image. Save the output in the folder with original MR image.

7. In the macOS command line, run c3d x.nii.gz -region 0x0x0vox 22x22x22vox -resample 200x200x200% -scale 0 -landmarks-to-spheres xx.txt 0.2 -o xxx.nii.gz where x.nii.gz is the image resulted from execution of 3D_to_1D in the step 2, xx.txt is the name of the text image resulted from Im2Ar(ImageName) in IgorPro command line in the previous step, xxx.nii.gz is the desired name of 44x44x44 LGN containing image representing edge-enhanced and upsampled version of the original LGN-containing image.

8. The shell script DSC calculates the Dice Similarity Coefficient between the delineation listed first in the text file (the input argument of the script), and all other listed delineations. For example, running ./DSC list.txt takes the segmentation listed first in the text file list.txt, and calculates its DSC with all other listed segmentations. The script DSC generates output text file that can be opened with Microsoft Excel.
